# Supplementary material for: WordCommentsAnalyzer: A windows software tool for qualitative research
Source: F1000Res. 2018 Sep 4;7:536. Originally published 2018 May 3. [Version 2] doi: 10.12688/f1000research.14819.2 (PMC6137411; doi:10.12688/f1000research.14819.2)
Supplement: Supplementary file 3 [file f1000research-7-17614-s0002.tgz › b2a79496-4ada-47c4-9682-a99ce867836d.zip!/Eight Iranian Health Journals/The Journal of Toloo-e-Behdasht_14.docx]

© The Journal of Toloo-e-Behdasht. This work is licensed under CC BY 4.0 (https://creativecommons.org/licenses/by/4.0/).

Volume & Issue: دوره 14 - شماره 6

Title: اثر مکمل‌یاری با کوآنزیم 10Q بر شاخص‌های التهابی(هموسیستئین و hs-CRP) و فشار خون در افراد مبتلا به سندرم متابولیک

http://tbj.ssu.ac.ir/article-1-1967-fa.html

مقدمه: سندرم متابولیک تجمع خوشه مانند گروهی از فاکتورهای قلبی عروقی شامل چاقی شکمی، دیس‌لیپیدمی، اختلال در متابولیسم گلوکز و پرفشاری خون می‌باشد که با افزایش فاکتورهای التهابی نیز همراه است. این مطالعه با هدف اثر مکمل کوآنزیم‌10Q بر فشار خون و شاخص‌های التهابی در بیماران مبتلا به سندرم متابولیک انجام شد. روش بررسی: طراحی این مطالعه به صورت کارآزمایی بالینی تصادفی شده دوسوکور بود که با شرکت 50 نفر بیمار مبتلا به سندرم متابولیک با طول مدت مکمل‌یاری سه ماهه انجام شد. بیماران به طور تصادفی به دو گروه آزمون و دارونما تقسیم شدند که گروه آزمون روزانه دو عدد سافت ژل 100 میلی‌گرمی کوآنزیم 10Q و گروه دارونما روزانه دو عدد سافت ژل مشابه فاقد مکمل دریافت کردند. قبل از مداخله و بعد از 12 هفته فشار خون، hs-CRP و هموسیستئین به عنوان شاخص‌های التهابی بیماران اندازه‌گیری شد. یافته‌ها: 45 نفر از افراد شرکت‌کننده، مطالعه را تا پایان ادامه دادند. کوآنزیم 10Q باعث کاهش فشار خون سیستولیک به میزان 66/0 سانتی‌متر جیوه(04/0=p) و کاهش فشار خون دیاستولیک به میزان 31/0 سانتی متر جیوه شد(001/0=p). در مورد فشار خون دیاستولیک اگر چه این تفاوت معنی‌دار بود، اما میانگین تغییرات آن نسبت به گروه دارونما تفاوتی نشان نداد. غلظت هموسیستئین در گروه مداخله از u/ml 09/2±97/11 به u/ml 93/1±31/10 (005/0p<) و hs-CRP از u/ml 46/3± 40/4 به u/ml 25/3± 64/3 (037/0=p) کاهش یافت. نتیجه‌گیری: این مطالعه نشان داد که مصرف روزانه 200 میلی‌گرم کوانزیم‌10Q به مدت 12 هفته در بیماران مبتلا به سندرم متابولیک منجر به کاهش معنی‌دار فشار خون سیستولی و دیاستولی و کاهش شاخص‌های التهابی در این بیماران می‌شود.

Title: بررسی رفتارهای خطرناک در رانندگان شهر یزد در سال 1392

http://tbj.ssu.ac.ir/article-1-1968-fa.html

مقدمه: نرخ تخلف رانندگی در ایران بسیار بالاست و تبعات ناگوار ناشی از آن باعث شده است تخلفات رانندگی یکی از مسائل اجتماعی مهم در کشور باشد. با توجه به افزایش تخلفات درسالهای اخیر در ایران، پژوهش حاضر تلاش کرده است رفتارهای خطرناک در رانندگی را مورد بررسی قرار دهد. روش بررسی: این مطالعه یک مطالعه توصیفی از نوع مقطعی بوده است.در این مطالعه جامعه هدف کلیه رانندگان وسایل نقلیه سبک تردد‌کننده در سطح شهر یزد بوده‌اند که مرتکب تخلف در رانندگی شده اند. حجم نمونه لازم جهت انجام مطالعه 300 نفر تعیین گردید. نحوه نمونه‌گیری به صورت تصادفی بود. جمع آوری داده‌ها با استفاده از پرسشنامه‌ای بود که طی یک مطالعه مقدماتی روایی و پایایی قسمت‌های مختلف آن به اثبات رسیده بود. داده‌ها پس از جمع‌آوری با استفاده از نرم‌افزار spss نسخه 18 و آزمون‌های آماری Anova، t-test، همبستگی پیرسون و رگرسیون مورد تجزیه و تحلیل قرار گرفت. یافته‌ها: افراد شرکت‌کننده در این مطالعه 288 نفر مرد و 12 نفر زن بودند. میانگین سنی افراد شرکت‌کننده 29/33 سال بود که 49 درصد افراد در گروه سنی 30-18 سال قرار داشتند. 73 نفر(3/24 درصد) افراد مجرد و227نفر(7/75 درصد) افراد متأهل بودند. بیشترین رفتارهای خطرناک انجام‌شده در حال رانندگی، صحبت با دیگر سرنشینان 6/73 درصد، 7/42 درصد استفاده از مواد خوراکی، 7/38 درصد نبستن کمربند و 7/36 درصد صحبت با موبایل، 4/27 درصد عدم رعایت فاصله طولی، 34 درصد سرعت غیر مجاز، 22 درصد سبقت غیر مجاز و 17درصد رانندگی در حال خستگی گزارش شده است. همچنین بین سن و انجام رفتارهای مخاطره آمیز در رانندگی ارتباط معنادار وجود داشت که با افزایش سن انجام رفتار خطرناک افزایش می‌یابد. بین تجربه رانندگی و انجام رفتار خطرناک نیز رابطه معنی‌دار وجود داشت. نتیجه‌گیری: گسترش فوق‌العاده تخلف رانندگی و تبعات زیان بار ناشی از آن باعث شده است این موضوع به عنوان یکی از مهم‌ترین مسائل اجتماعی کشور مورد توجه قرار‌گیرد. براین اساس پیشنهاد می‌شود تخلف رانندگی به عنوان یک مسئله اجتماعی بیش‌تر مورد توجه محافل علمی کشور قرار‌گیرد. با توجه به نتایج مطالعه حاضر پیشنهاد می‌شود که یک برنامه‌ریزی اصولی جهت آموزش صحیح افراد جهت آشنایی با قوانین راهنمایی و رانندگی انجام شود تا کمتر شاهد تخلفات رانندگی باشیم.

Title: بررسی ارتباط شرایط محیط کار دندانپزشکان با کیفیت زندگی آنان در سال 1393

http://tbj.ssu.ac.ir/article-1-1971-fa.html

مقدمه: در مطالعات مختلف مربوط به مشاغل، این نکته دریافت شد که استرسهای وابسته به شغل در بین دندانپزشکان شیوع بالایی دارد. این استرس‌ها که می‌تواند ناشی از عواملی چون صدا و روشنایی نامناسب باشد سبب ناراحتی عاطفی و به خطر افتادن سلامت فیزیکی گردیده و کیفیت زندگی آنان را تحت تاثیر قرار می‌دهد. با توجه به اهمیت موضوع، مطالعه‌ای با هدف بررسی کیفیت زندگی کاری دندانپزشکان، دو مولفه مهم صدا و روشنایی و استرس شغلی در این حرفه ارزیابی شده است. روش بررسی: در این مطالعه تحلیلی، توصیفی، مقطعی محقق با مراجعه به مطب دندانپزشکان، مقدار روشنایی و صدای محیط کار را اندازه‌گیری و کیفیت زندگی آنان را با استفاده از پرسشنامه کیفیت زندگی مک‌گیل و استرس را با پرسشنامه ارزیابی استرس سنجیده و ارتباط بین متغیرهای کمی با استفاده از رگرسیون برای مدل‌سازی نیز  از رگرسیون چندگانه استفاده گردید. یافته‌ها: میانگین میزان صدای موضعی مته دندانپزشکی درمطب‌های خصوصی و دولتی به ترتیب 5/75 و 4/77 و روشنایی موضعی در 2/2 درصد یونیت‌های دندانپزشکی درمانگاه‌ها وکلنیک‌ها، زیر حد استاندارد بود. یافته‌های این مطالعه نشان می‌دهد که 9/58 درصد از دندانپزشکان شرکت‌کننده در مطالعه کیفیت زندگی خود را مطلوب و نسبتاً مطلوب می‌دانند. نتیجه‌گیری: همه این موارد حکایت از اثرشرایط محیط کار بر استرس و کیفیت زندگی دارد که با آموزش راه‌های کنترل و مدیریت آن می‌توان از اثرات تخریبی این اختلال روانشناختی تا حد قابل ملاحظه ای کاست و کیفیت زندگی را افزایش داد.

Title: بررسی میزان مراجعه بیماران برای انجام درمانهای پیشگیرانه پس از درمان جراحی لثه

http://tbj.ssu.ac.ir/article-1-1972-fa.html

مقدمه: درمان حمایتی پریودنتال از پیشرفت و برگشت بیماری پریودنتال در میان بیمارانی که قبلا مبتلا به پریودونتیت بوده‌اند و درمان شده‌اند جلوگیری می‌کند. همچنین باعث کاهش انسیدانس از دست دادن دندان و افزایش امکان شناخت درمان سایر بیماری‌ها یا شرایطی که حاصل بیماری دهانی هستند، می‌شود. لذا مطالعه حاضر با هدف تعیین فراوانی مراجعه بیماران جهت ارزیابی وضعیت لثه خود پس از درمان جراحی لثه در یکی از مطب‌های تخصصی پریو در شهر یزد در سال 1390 انجام شد. روش بررسی: جهت انجام این مطالعه 356 پرونده‌ بیمارانی که در مطب خصوصی طی سال‌های 1375 تا 1384، تحت درمان جراحی لثه قرار گرفته بودند بصورت تصادفی انتخاب شده و دفعات مراجعه بعد از جراحی که صرفاً به منظور معاینه انجام گرفته بود و مشخصات دموگرافیک افراد در چک لیستی ثبت گردید. داده‌ها با استفاده از نرم افزار آماری SPSS نسخه 16 با استفاده از آمار توصیفی مورد تجزیه و تحلیل قرار گرفتند. یافته‌ها: پرونده‌های مورد مطالعه، متعلق به 205 زن(%6/57) و 151 مرد(%4/42) بودند. بیماران در رده سنی 67-14 سال با میانگین سنی 48/9±80/37 بودند. 281 بیمار (%78/93) جهت ارزیابی وضعیت لثه خود پس از درمان جراحی لثه اصلاً مراجعه ننموده بودند. 42 نفر (79/11%) تنها یکبار پس از جراحی مراجعه نموده و 22 نفر (17/6%) دو بار مراجعه کرده بودند. تعداد کمی از بیماران 3 بار (68/1%) و بیشتر مراجعه نموده بودند. ارتباط آماری معنی‌داری بین میزان مراجعه پس از جراحی لثه و سن، جنس، میزان تحصیلات و شغل بیماران وجود نداشت. نتیجه‌گیری: علی رغم اهمیت درمان‌های نگهدارنده پس از جراحی لثه بیش از 90 درصد از بیمارانی که تحت جراحی لثه قرار گرفته بودند، هیچ دفعه جهت دریافت درمان‌های حمایتی مراجعه نکرده بودند. لذا تاکید و تشویق پریودنتیست جهت مراجعه منظم بیماران، برای اطمینان از حفظ نتایج، جلوگیری از پسرفت نتایج درمان

Title: بررسی روند حوادث ترافیکی و مرگ‌و‌میر ناشی از آن در استان یزد طی سال‌های1390-1385

http://tbj.ssu.ac.ir/article-1-1973-fa.html

مقدمه: حوادث ترافیکی دومین علت مرگ پس از بیماری‌های قلبی عروقی در کشور ایران(1)و سومین علت مرگ بعد از بیماری‌های قلبی عروقی و سرطان در کشور‌های در حال توسعه حوادث ترافیکی است(2). در کشور ما حدود 25000 نفر در سال و 70 نفر در روز بر اثر حوادث ترافیکی فوت کرده‌اند(3). با توجه به شیوع روز افزون جمعیت و وسایل نقلیه و شیوع بالای حوادث ترافیکی در کل کشور و استان یزد، این مطالعه بر آن است تاروند مرگ‌و‌میر ناشی از حوادث ترافیکی را در یک دوره 5 ساله بررسی نموده و با آمار قبلی مقایسه نماید. روش‌بررسی: این مطالعه به روش گذشته‌نگر مبتنی بر اطلاعات نظام ثبت سوانح می‌باشد که با استفاده از آمار سال‌های1390-1385 مرکز مدیریت حوادث و فوریت‌های پزشکی یزد، پزشکی قانونی و پلیس راهور انجام گردید. حجم نمونه به روش سرشماری است و کلیه موارد مرگ‌و‌میر ناشی از حوادث ترافیکی را شامل می‌شود(2492 نفر). داده‌ها از فرم‌های ثبت اطلاعات مصدومین و متوفیان جمع آوری گردیده و پس از گردآوری دادها با استفاده از نرم افزار SPSS نسخه 16 مورد تجزیه تحلیل قرار گرفت. روند مرگ‌و‌میر ناشی از حوادث ترافیکی بر اساس مدل رگرسیون پوآسون گزارش شد. یافته‌ها: باتوجه به بررسی موارد مرگ‌و‌میر ناشی از حوادث ترافیکی میزان بروز مرگ‌و‌میر از7/4 درصد در سال 1385 به 37/3 درصد در سال 1390 کاهش داشته است. اکثریت موارد فوتی ناشی از ضربه به سر می‌باشد که میزان آن از 9/79 درصد در سال 1385 به 3/62در سال 1390 کاهش داشته است. نتیجه‌گیری: روند سوانح رانندگی رو به کاهش است. احتمالا اجرای قوانین و مقررات رانندگی از یک سو و فرهنگ‌سازی از سوی دیگر از جمله عوامل کاهش روند تصادفات در طول سال‌های 1385 تا 1390 می‌باشد.

Title: بررسی عوامل موثر بر سلامت اجتماعی معلمان مقطع ابتدایی ناحیه یک شهر یزد

http://tbj.ssu.ac.ir/article-1-1974-fa.html

مقدمه: امروزه نظام آموزش و پرورش جایگاه ویژه‌ای در تأمین سلامت اجتماعی افراد جامعه دارد. مسلماً کمبود سلامت اجتماعی در نیروهای آموزشی این نظام می‌تواند پیامدهای نامناسبی بر انسجام و مشارکت نسل­های آینده و سلامت جامعه داشته باشد. لذا با توجه به اهمیت مسئله، این مطالعه به بررسی میزان سلامت اجتماعی معلمان و عوامل مؤثر برآن می­پردازد. روش بررسی: این تحقیق بصورت پیمایش و با استفاده از پرسشنامه‌ سلامت اجتماعی کینز انجام شد. حجم نمونه 300 نفر از آموزگاران، مربیان و عوامل اداری مدارس ابتدایی ناحیه یک شهریزد هستند که به­صورت نمونه‌گیری خوشه‌ای یک مرحله‌ای ساده انتخاب شدند. داده‌های به دست آمده توسط نرم‌افزار SPSS نسخه 19 تحلیل شدند. یافته‌ها: میزان سلامت اجتماعی پاسخگویان بالاتر از حد متوسط، پذیرش اجتماعی پاسخگویان کمترین و مشارکت اجتماعی آنان بیشترین میزان است. سلامت اجتماعی با محل تولد، درآمد، مسکن شخصی، تعداد اعضای خانوار، رضایت زناشویی و شغلی، سلامت سازمانی مدرسه، معدل دانش آموزان، اعتقاد به سلامت جامعه، دینداری، حمایت اجتماعی و عاطفی رابطه مثبت و معنی‌داری دارد. بین سن، جنس، محل سکونت، تحصیلات، سن، رضایت اوقات فراغت، استفاده از وسایل نوین اطلاعاتی، سمت شغلی، نوع مدرسه و تعداد دانش‌آموزان با سلامت اجتماعی رابطه معنی‌داری مشاهده نشد. در مدل رگرسیونی نیز متغیرهای دینداری، اعتقاد به سلامت جامعه و حمایت اجتماعی 43/0 درصد از تغییرات سلامت اجتماعی را تبیین می‌کنند. نتیجه‌گیری: افزایش امکانات مدارس، افزایش رضایت شغلی معلمان باایجاد امنیت شغلی، امکانات مشاوره خانوادگی، حمایت مالی و تسهیلات مناسب برای کارمندان محتاج می‌تواند به بهبود سلامت اجتماعی معلمان یاری رساند.

Title: بررسی اثر مصرف خوراکی دو مقدار مختلف بذر کتان بر شاخص‌های تن سنجی و استرس اکسیداتیو درافراد مبتلا به پیش دیابت دارای اضافه وزن وچاق:یک کارآزمایی بالینی شاهددار تصادفی

http://tbj.ssu.ac.ir/article-1-1975-fa.html

مقدمه: چاقی وافزایش استرس اکسیداتیو منجر به افزایش مقاومت انسولینی می‌گردد. استفاده از بذر کتان به صورت روز افزون در رژیم افراد، به منظور کاهش وزن افزایش یافته است. داده‌های کمی در خصوص اثر بخشی بذر کتان بر بهبود شاخص‌های تن سنجی واسترس اکسیداتیو وجود دارد. هدف ازمطالعه حاضر بررسی اثر مصرف خوراکی دو مقدار مختلف بذر کتان بر شاخص‌های تن‌سنجی واسترس اکسیداتیو در افراد مبتلا به پیش دیابت دارای اضافه وزن وچاق بوده است. روش بررسی: در این کارآزمایی بالینی شاهددار تصادفی، 99 فرد مبتلا به پیش دیابت با نمایه توده بدنی 25 تا 9/34 کیلوگرم بر مترمربع به شکل تصادفی به سه گروه تقسیم شدند. دو گروه دریافت‌کننده بذر کتان به میزان 40 گرم درروز (گروهHD) و 20 گرم درروز (گروه LD)، بذر کتان آسیاب‌شده را به مدت 12 هفته دریافت کردند و گروه سوم به عنوان گروه غیرآزمون (C) هیچ مداخله‌ای دریافت نکردند. شاخص‌های آنتروپومتری وغلظت مالون دی‌آلدهید قبل وبعداز مطالعه اندازه‌گیری شد. یافته‌ها: 92 نفر از بیماران مداخله 12 هفته‌ای را به اتمام رساندند. در پایان مطالعه وزن، میانگین توده بدنی ودور کمر در گروه‌های دریافت‌کننده بذر کتان نسبت به گروه غیر آزمون، کاهش معنی‌داری داشت(005/0>p). اما میانگین غلظت مالون‌دی‌آلدهید به عنوان شاخص استرس اکسیداتیو در گروه  HDو گروه C کاهش معنی‌داری در پایان مطالعه داشت، اما این تفاوت در بین گروه‌های مورد مطالعه معنی‌دار نبود. نتیجه‌گیری :نتایج این مطالعه نشان داد که مصرف بذر کتان به میزان‌های 20 و40 گرم در روز می‌تواند شاخص توده بدنی و اندازه دور کمررا در افراد در مرحله پیش دیابت کاهش دهد، اما تاثیر معنی‌داری بر استرس اکسیداتیو در این بیماران ندارد.

Title: بررسی عوامل موثر بر اتخاذ رفتارهای ارتقاء‌دهنده سبک زندگی نوجوانان با کاربرد تئوری رفتار برنامه‌ریزی شده

http://tbj.ssu.ac.ir/article-1-1976-fa.html

مقدمه: سبک زندگی ناسالم باعث بوجود آمدن خطرات بهداشتی مهم در نوجوانی و بیماری‌های غیرواگیر در بزرگسالی می‌شود. ارزیابی نیازهای آموزشی با استفاده از تئوری  قبل از مداخله یک پایه و اساس برای برنامه‌ریزی آموزشی در ارتقای سبک زندگی است. هدف مطالعه حاضر بررسی عوامل موثر بر اتخاذ رفتارهای ارتقاء‌دهنده سبک زندگی دانش‌آموزان شهرگناباد با کاربرد تئوری رفتار برنامه ریزی شده بود. روش بررسی: در این مطالعه توصیفی تحلیلی، 175 دانش آموز در محدوه سنی 15-12 سال با روش نمونه‌گیری چند‌مرحله‌ای تصادفی انتخاب شدند. برای جمع‌آوری داده‌ها پرسشنامه طراحی‌شده بر اساس سازه‌های تئوری رفتار برنامه‌ریزی شده در زمینه سبک زندگی نوجوانان استفاده شد. داده‌ها در نرم‌افزارspss  نسخه 20 با استفاده از ضریب همبستگی پیرسون، آنالیز رگرسیون چند گانه و آزمون مستقل t-test تجزیه و تحلیل شدند. یافته‌ها: میانگین و انحراف‌معیار (9/0± 5 /13) سال به دست آمد. یافته‌ها نشان دادند کیفیت سبک زندگی با افزایش سن دانش‌آموزان و تحصیلات پدر کاهش می‌یابد. در تغذیه سالم (438/0=P) و عدم پذیرش سیگار و الکل (256/0=P) قصد با رفتار ارتباط نشان نداد. نگرش پیش بینی قابل توجهی برای قصد (268/0=B) و رفتار ( 638/0=B) تغذیه سالم بود. در فعالیت بدنی قصد توسط کنترل رفتار درک‌ شده (244/0=B) و رفتار توسط هنجارهای ذهنی پیش‌بینی شد (424/0=B). در مدیریت استرس قصد با هنجارهای ذهنی (455/0=B) و رفتار باکنترل رفتار درک شده پیش‌بینی شد (767/0=B). در عدم پذیرش سیگار و الکل، نگرش ارتباط قوی با قصد (829/0=B) و کنترل رفتار درک‌شده ارتباط قوی با رفتار نشان داد (311/0=B). نتیجه‌گیری: فقدان ارتباط بین قصد و رفتار نشان می‌دهد که عوامل جمعیت‌شناختی بجز قصد ممکن است رفتار در نوجوانان را تحت‌تاثیر قرار دهد و مداخلات آموزشی با تمرکز بر سازه کنترل رفتار درک‌شده به نظر می‌رسد راه مهمی برای رسیدن به رفتار مورد نظر باشد. رفتار فعالیت بدنی تحت‌تاثیر مستقیم ساختارهای اجتماعی قرار گرفت. بنظر می‌رسد ارتقای رفتارهای ورزشی در این گروه سنی با همکاری خانواده موفق‌تر باشد.

Title: اندازه‌گیری غلظت گاز رادون در معادن زیرزمینی سرب و روی استان یزد

http://tbj.ssu.ac.ir/article-1-1977-fa.html

مقدمه:گاز رادون با حدود دو سوم تابش زمینه، بیشترین منبع پرتودهی جامعه بوده و مواجهه با دختران آن با تابش آلفا احتمال ریسک سرطان ریه را افزایش می‌دهد بطوری‌که در امریکا پس از سیگار دومین عامل بروز سرطان ریه است. گرچه بررسی میزان رادون در اروپا بیش از کشورهای جهان سوم انجام گرفته ولی مطالعه میزان رادون به تدریج در سراسر دنیا در معادن رو به افزایش است که می‌توان به مطالعه غیاسی، ایشیموری فیزن و کوبال در معدن سرب اشاره کرد، با توجه به اهمیت موضوع و وجود تعداد قابل توجه معادن زیرزمینی در استان یزد و کارگران شاغل در این معادن میزان گاز رادون معادن سرپوشیده سرب و روی مورد ارزیابی قرار گرفت. روش بررسی: در یک مطالعه میدانی و به روش توصیفی- مقطعی میزان رادون معادن سرب و روی استان یزد را با روش اکتیو و با استفاده از دستگاه RAD7 با رنج اندازه گیری آن 400.000- 4 بکرل بر متر مکعب در فصل تابستان، مورد سنجش و میزان ذرات آلفا ایجاد شده از گاز رادون مورد شمارش قرار گرفت. یافته‌ها: با توجه به نتایج مطالعه، در معادن 1 و2، سه و در معدن 1، دو ایستگاه، رادون سنجی انجام گرفت. که بیشترین غلظت در معدن 3 با مقدار3/4183 بکرل بر متر مکعب و کمترین غلظت در معدن 1 با مقدار 57 بکرل برمتر مکعب بود، و میزان میانگین غلظت گاز رادون در معادن 1، 2 و 3 به ترتیب برابر با 5/848، 83/125 و 4/1667 بکرل بر متر مکعب اندازه‌گیری گردید. همچنین میانگین غلظت گاز رادون در محل کار کارگران 3/2068، در محل رفت و آمد کارگران 9/219 و در محل استراحت و محیط خارج از معدن 75/19 بکرل بر متر مکعب اندازه‌گیری شد. از طرفی مقدار همبستگی بین غلظت گاز رادون با عیار سرب معدن نیز 899/0- می‌باشد. نتیجه‌گیری: نتایج حاصل از این مطالعه نشان می‌دهد که، غلظت گاز رادون سنجش شده در 5/62 درصد نقاط اندازه‌گیری‌ شده کمتر از حد اقدام، 5/12درصد نقاط بین حد اقدام و حداکثر مقدار مجاز توصیه‌شده و 25 درصد بیشتر از حداکثر مقدار مجاز توصیه‌شده می‌باشد. همچنین محل و ایستگاه اندازه‌گیری مهمترین عامل در غلظت گاز رادون می‌باشد.

Title: بررسی کمیت و کیفیت زباله تولیدی شهر سیرجان از زمستان 1392 تا پاییز 1393

http://tbj.ssu.ac.ir/article-1-1978-fa.html

مقدمه: نرخ تولید زباله و ترکیبات فیزیکی آن به عوامل متعددی بستگی دارد که این عوامل در مناطق مختلف فرق داشته و به این علت کمیت و کیفیت زباله در شهرهای مختلف با هم اختلاف دارند. در تحقیق حاضر کمیت و کیفیت زباله تولیدی شهر سیرجان مورد بررسی قرار گرفت. روش بررسی: مطالعه توصیفی مقطعی است که از زمستان 1392 تا پاییز 1393در مرکز تحیقات مهندسی بهداشت محیط دانشگاه علوم پزشکی کرمان انجام شد. در هر فصل ماه دوم انتخاب و سپس در هفته دوم یا سوم هر ماه، چهار روز وسط هفته انتخاب گردید. هر روز ده نمونه تصادفی برداشت و هر نمونه 5/0 متر مکعب حجم داشت. اجزاء تشکیل دهنده مواد زائد در 9 دسته تقسیم بندی شد. در هر فصل 40 نمونه و مجموعا در چهار فصل 160 نمونه مورد آنالیز قرار گرفت و میزان تولید زباله شهر سیرجان محاسبه گردید. تجزیه و تحلیل داده ها با استفاده از آمار توصیفی انجام شد. یافته ها: میانگین تولید روزانه زباله شهر سیرجان در سال 75/150 تن بود. بیشترین میزان تولید زباله در فصل پاییز به میزان 158 تن در روز بود. بیشترین میانگین جزء تشکیل دهنده زباله مواد فساد پذیر به میزان 2/68 درصد و کمترین،  بطری PET به میزان 3/1 درصد بود. بررسی های میدانی و اطلاعات مشهود نشان داد که محل فعلی دفن زباله های شهر سیرجان نامناسب است. نتیجه گیری: با توجه به میزان بالای مواد فسادپذیر در زباله های تولیدی توصیه می شود تولید کمپوست مورد توجه قرار گیرد و سپس تمهیدات لازم جهت راه اندازی خط بازیافت کاغذ و مقوا و مواد دارای ارزش تجاری در دستور کار قرار گیرد. تعیین محل جدیدی برای دفن زباله های شهر از اولویت های اساسی است

Title: مقایسه الگوی رشد 5 سال ابتدای زندگی کودکان مبتلا به کم‌کاری مادرزادی تیروئید شهر یزد با الگوی رشد کودکان سالم سازمان بهداشت جهانی در سال1393 :یک مطالعه کوهورت

http://tbj.ssu.ac.ir/article-1-1979-fa.html

چکیده مقدمه: کم‌کاری مادرزادی تیروئید یکی از عمده‌ترین علل قابل پیشگیری و درمان اختلالات رشد جسمی می‌باشد.لذا پژوهش حاضر با هدف بررسی توصیفی الگوی رشد 5 سال ابتدای زندگی کودکان مبتلا به کم‌کاری مادرزادی تیروئید شهر یزد با توجه به الگوی رشد کودکان سالم سازمان بهداشت جهانی انجام شد. روش بررسی: پژوهش حاضر به صورت کوهورت گذشته نگر (Retrospective  cohort) بوده که با ماهیت توصیفی بر روی کلیه نوزادان متولد سال‌های 1385 تا 1387 که توسط طرح غربالگری به عنوان بیمار تشخیص داده شده بودند، پس از لحاظ نمودن معیارهای ورود و خروج  انجام شد، سپس الگوی رشد قد، وزن و دورسر 5 سال ابتدای زندگی این کودکان با توجه به الگوی رشد کودکان سالم سازمان بهداشت جهانی به تفکیک سن و جنس در صدکهای 3، 15، 50، 85 و97 مورد بررسی و مقایسه قرار گرفت. یافته‌ها: الگوی رشد قد،وزن و دورسر دختران مبتلا به بیماری به موازات الگوی رشد قد،وزن و دورسر دختران سالم سازمان بهداشت جهانی پیش‌رفته بود .الگوی رشد قدی پسران بیمار پس از 36ماهگی، الگوی رشد وزنیشان بعد از 9 ماهگی و الگوی رشد دورسر آنها در سن 24 ماهگی شبیه الگوی رشد پسران سالم سازمان بهداشت جهانی شده‌بود. نتیجه‌گیری: الگوی رشد کودکان مبتلا به کم‌کاری مادرزادی تیروئید پس از درمان و ادامه مراقبت‌های پزشکی به سمت الگوی رشد طبیعی پیش خواهد رفت.

Title: بررسی ارتباط استرس و همبسته‌های آن با بیماری عروق کرونر قلب در بیماران مراجعه‌کننده به بیمارستان‌های شهر یزد- 1391

http://tbj.ssu.ac.ir/article-1-1980-fa.html

مقدمه: بیماری قلبی- عروقی یکی از مهمترین عوامل مرگ‌و‌میر در جهان است. در سال‌های اخیر نقش عوامل روانی در بروز و تشدید بیماری‌های قلبی- عروقی بویژه عروق کرونر، بیش از پیش مورد توجه قرار گرفته است. بنابراین هدف از این مطالعه تعیین میزان استرس درک‌شده در بیماران عروق کرونری می‌باشد تا گامی در جهت رفع برخی مشکلات بیماران قلبی و کاهش هزینه‌های اضافی برداشته شود. روش بررسی: در این مطالعه مورد-شاهدی، 250 بیمار مبتلا به بیماری عروق کرونری که در بیمارستان‌های افشار و شهید صدوقی یزد بستری شده بودند با 250 فردی که با تشخیص متخصصین قلب، از لحاظ قلبی سالم بودند مقایسه گردیدند. دو گروه که از نظر سن و جنس همسان شده‌بودند، با استفاده از پرسشنامه‌ی استرس هولمز- راهه، میزان استرس و عوامل استرس‌زای این دو گروه مورد مقایسه قرار گرفت. آزمون‌های  T-test، کای دو، من- ویتنی و رگرسیون لوجستیک برای مقایسه‌ی متغییرها و نمره‌ی استرس دو گروه انجام گردید. از نرم افزار16 SPSS برای تجزیه و تحلیل داده‌ها استفاده شد. یافته‌ها: یافته‌ها نشان داد، بیماران مبتلا به انفارکتوس قلبی، طی یک سال قبل از وقوع حمله قلبی، رویدادهای استرس‌زای روانی -اجتماعی بیشتری را در مقایسه با افراد گروه شاهد تجربه کرده بودند. 4/16% موردها و 6/1% شاهدها، سطح استرس بیش  از 300واحد داشتند(001/0p<). شانس ابتلا به بیماری عروق کرونری در این سطح از استرس 20 برابر گروه مشابه شاهد بود. همچنین مهمترین حوادث استرس‌زا که توسط افراد مورد مطالعه درک شده بود، تغییر در وضعیت اقتصادی، زخمی یا بیمارشدن و تغییر ساعات خواب دریک سال گذشته بود. نتیجه‌گیری: در مطالعه انجام شده، بیماران قلبی در مقایسه با افراد سالم در مواجهه با عوامل استرس‌زای بیشتری بودند که علاوه بر اثر مستقیم بر بروز و تشدید بیماری قلبی، سیکل معیوبی در روند بهبودی بیماران قلبی ایجاد می‌کرد. بنابراین در توانبخشی قلبی می‌توان بر نقش استرس بیش از پیش تاکید نمود.

Title: بهبود کیفیت بهداشتی و فیزیکوشیمیایی پنیر با استفاده از اسانس روغنی گیاه کلپوره

http://tbj.ssu.ac.ir/article-1-1981-fa.html

چکیده افزایش تقاضا برای مصرف غذاهای سلامت‌بخش با خصوصیات حسی مطلوب موجب نوآوری و توسعه محصولات جدید در صنعت غذایی در سراسر دنیا شده است، بنابراین در مطالعه حاضر تاثیر غلظت‌های مختلف اسانس روغنی کلپوره بر خصوصیات شیمیایی و ارگانولپتیکی پنیر سفید ایرانی بررسی شده است. روش بررسی: خصوصیات کیفی (پروتئین، چربی، نمک و pH) پنیر سفید ایرانی فرآوری‌شده با غلظت‌های مختلف اسانس گیاه کلپوره (15، 30 و 60 ppm) طی دوره رسیدن دو ماهه طی فواصل زمانی 15 روز به همراه خواص حسی نمونه‌ها مورد بررسی قرار‌گرفت. در طول رسیدن پنیر کاهش تدریجی و پایدار مقادیر pH تمامی نمونه‌ها مشاهده شد. همچنین نمونه‌های پنیر دارای اسانس از مقادیر pH پائین‌تری در مقایسه با پنیر کنترل برخوردار بودند، به گونه‌ای که در انتهای دوره نگهداری پنیر کمترین میزان pH (38/3) مربوط به تیمار پنیر واجد 30  ppm اسانس بود. بر اساس یافته‌های این مطالعه در تمام نمونه‌های پنیر در طول رسیدن پنیر درصد رطوبت به طور معنی‌داری کاهش و درصد پروتئین و نمک افزایش معنی‌داری را نشان داد (05/0>P). اما درصد چربی بر پایه ماده خشک از تغییرات معنی‌داری برخوردار نبود. کمترین میزان رطوبت (42/62 درصد) در انتهای دوره ارزیابی در تیمار پنیر واجد اسانس 30 ppmمشاهده گردید. یافته‌های ارزیابی حسی نمونه‌ای مختلف پنیر نشان داد که بهترین غلطت اسانس از نظر تولید پنیر با خواص ارگانولپتیکی مطلوب غلظت30 ppm بود. بر اساس یافته‌های حاصل از این مطالعه می‌توان از اسانس گیاه کلپوره در کنار سایر روش‌های محافظتی در جهت کاهش کاربرد نگهدارنده‌ها و طعم‌دهنده‌های سنتتیک و تولید تولید فراورده غذایی با خصوصیات طعمی جدید بویژه محصولات لبنی پر مصرف همچون پنیر بهره جست.

Title: ارزیابی زیست‌محیطی (EIA) و مکان‌یابی بهینه محل دفن پسماند جامد شهری با استفاده از روشSAW، GIS و ماتریس لئوپولد (مطالعه موردی شهر یزد)

http://tbj.ssu.ac.ir/article-1-1983-fa.html

چکیده مقدمه: تولید پسماند از هر منشا به مفهوم تولید انواع آلاینده‌ها است. هرگونه طرح غیر اصولی برای دفع این مواد و اصولاً وجود این مواد دارای اثرات نامطلوب زیست‌محیطی می باشد. از این رو برای مدیریت بهداشت شهری ارزیابی اثرات مثبت و منفی پروژه‌های مهندسی- بهداشتی مؤثر بر محیط‌زیست مانند احداث لندفیل، امری ضروری است. روش بررسی: این مطالعه با هدف مکان‌یابی و ارزیابی اثرات زیست‌محیطی(EIA: Environmental Impact Assessment مکان دفن پسماند جامد شهری (MSW: Municipal Solid Waste)یزد با استفاده از سیستم اطلاعات جغرافیایی انجام گرفته است. به همین منظور، لایه‌های مورد نیاز این روش تهیه شد تا طی 3 مرحله شامل: مکان یابی مناطق مستعد دفن، بازدید صحرایی جهت بررسی خصوصیات مناطق مستعد و انجام ارزیابی زیست‌محیطی با استفاده از ماتریس لئوپولد جهت رتبه‌بندی مناطق و انتخاب بهینه‌ترین مکان، جایگاه مناسب تعیین گردد. یافته‌ها: با استفاده از لایه‌های متعدد از قبیل: زمین شناسی، کاربری اراضی، فاصله از محدوده شهر، شیب، فاصله از جاده، پوشش گیاهی، نفوذپذیری و هیدرولوژی، مکان­یابی و وزن­دهی به این لایه ها با روش ساده افزایشی (SAW: Simple additive weighting) صورت گرفت. در نتیجه 4 جایگاه در رده مناسب قرار گرفتند. نتیجه‌گیری: با استفاده از روش ماتریس لئوپولد (Matrix Leopold) منطقه 1(با مختصات مرکزی طول: 256915 متری و عرض: 3540127 متری) به عنوان مکان بهینه جهت دفن بهداشتی‌- مهندسی مشخص گردید. این مقاله حاصل بخشی از پایان­نامه مقطع کارشناسی ارشد می­باشد.

Title: شناسایی میکروارگانیسم‌های غالب بیوفیلتر چکنده در تصفیه هوای آلوده به فرمالدوئید و اتانول

http://tbj.ssu.ac.ir/article-1-1982-fa.html

مقدمه: شناسایی باکتری‌های تجزیه‌کننده مواد سمی، گام مهمی در روند تکامل سیستم‌های تصفیه هوا می‌شود. باکتری‌های موثر در تصفیه و حذف آلاینده‌ها متناسب با نوع آلاینده و نوع سیستم و شرایط محیطی حاکم بر آن متفاوت است. با شناسایی این باکتری‌ها می‌توان شرایط بهینه برای عملکرد سیستم را تعیین و به حداکثر راندمان دست پیدا کرد و با استفاده از روش‌های بیوتکنولوژی نسبت به تقویت آنها برای تصفیه آلاینده‌ها مورد محسوب نظر اقدام نمود. هدف اصلی از انجام این مطالعه بررسی میکروارگانیسم‌های غالب در دو بیوفیلتر چکنده می‌باشد که در یکی از فرمالدوئید و در دیگری از اتانول به عنوان تنها منبع کربن استفاده گردید. روش بررسی: در این مطالعه دو پایلوت بیوفیلتر چکنده در مقیاس آزمایشگاهی ساخته شده است. پس از تلقیح میکروارگانیسم‌ها و طی یک دوره سه ماهه سازگار سازی، این بیوفیلترها مورد مطالعه طولانی مدت قرار گرفتند. پس از پایان آزمایشات با نمونه گیری از بیوفیلم‌ها و انجام مطالعات میکروبیولوژی باکتری‌های غالب در بیوفیلتر شناسایی و گزارش شدند. یافته‌ها: در این مطالعه محققین موفق به یافتن میکروارگانیسم‌های سالمونلا بونگوری، پنومونیا سوبسپسیس، پنومونیا کلبسیلا و... . در بخشهای مختلف بیوفیلترها شدند. نتیجه‌گیری: راهبری راکتورهای بیولوژیکی مختلف در شرایط گوناگون می‌تواند منجر به تغییر وسعی در گونه‌های میکروبی موجود در آنها شود. شناسایی میکروارگانیسم‌های فعال در هر راکتور می‌تواند منجر به یافتن شرایط بهینه و یا خطرات ناشی از انتقال میکروارگانیسم‌های موجود در راکتور به بدن انسان شود.

Title: جداسازی و شناسایی بیوشیمیایی باکتریهای اسید لاکتیک با پتانسیل پروبیوتیکی در ماست های محلی استان یزد

http://tbj.ssu.ac.ir/article-1-1985-fa.html

مقدمه: استفاده روز افزون از فرآورده های لبنی صنعتی به جای سنتی ممکن است منجر به افزایش حذف باکتریهای پروبیوتیک شود. لذا این مطالعه به منظور جداسازی و شناسایی بیوشیمیایی باکتریهای اسید لاکتیک با پتانسیل پروبیوتیکی در ماست های محلی استان یزد انجام گرفت. روش بررسی : 96 نمونه ماست (32 نمونه ماست بز،32 نمونه ماست میش و32 نمونه ماست گاو) از منـاطق مختلف استان یزد جمع آوری شد. نمونه­های ماست ابتدا در محیط broth MRS غنی سازی و سپس بر روی بر روی محیطagar MRS کشت داده شدند. برای شناسایی اولیه باکتری­های اسید لاکتیک از تست­های گرم وکاتالاز وجهت بررسی پتانسیل پروبیوتیکی باکتری­های اسید لاکتیک شناسایی شده ابتدا از تست­ مقاومت به اسید وسپس از تست مقاومت به املاح صفراوی استفاده شد.در ادامه شناسایی گونه­ باکتری­های اسید لاکتیک توسط تست­های بیوشیمیایی در دمای 15و 45 درجه سانتیگراد، هیدرولیز آرژنین و تولید گاز از گلوکز انجام گرفت. یافته ها : ازمجموع 96 نمونه ماست جمع آوری شده،75 نمونه مثبت بدست آمد،که بر اساس تست­های فنوتیپی و بیوشیمیایی 47 باکتری اسید لاکتیک شناسایی شدند. از این 47 ایزوله لاکتیک، 24 ایزوله دارای مقاومت به اسید و در مرحله بعد،12 ایزوله مقاومت به صفرا را داشتند. از میان 12 ایزوله با توان پروبیوتیکی، 5 ایزوله کوکسی از گونه پدیوکوکوس اسیدلاکتیسی و 7 ایزوله از جنس لاکتوباسیلوس که متعلق به گونه­های ل. پلانتاروم، ل. برویس، ل. فرمنتوم و ل.کفیر بودند. نتیجه گیری: یافته های حاصل از این مطالعه نشان از تنوع بالای اسیدلاکتیک باکتری ها در ماست های بومی استان یزد دارد.

Title: کاربرد گرانول پوست گردو بعنوان جاذب طبیعی در حذف نیکل از محلولهای آبی

http://tbj.ssu.ac.ir/article-1-1984-fa.html

چکیده مقدمه: افزایش آلودگی محیط زیست توسط فلزات سنگین سبب نگرانی‌های بسیار جدی بدلیل خصوصیات سرطانزایی، تجزیه ناپذیری و تجمع بیولوژیکی آنها شده است؛ نیکل یکی از عمومی ترین آلاینده های دارای تقدم در محیط زیست بوده که حذف آن حائز اهمیت فراوانی است. روشهای مختلف و متعددی برای حذف و جداسازی یون‌های فلزات سنگین از محلول‌های آبی وجود دارد.  اغلب این روشها در غلظت‌های بالای فلزات کاربرد دارند و دارای معایبی از قبیل گرانی دستگاه‌ها و عملیات، تولید لجن یا سایر مواد زائد سمی، انرژی و فضای زیاد می باشند. از میان این روش ها، فرآیند جذب؛ به دلیل اینکه روشی ساده، کم هزینه و مؤثر برای حذف یونهای  فلزات سنگین در غلظت‌های کم و متوسط است، توجه علاقمندان زیادی را به خود جلب کرده است.هدف از این مطالعه بررسی کارآیی جذب یون نیکل از محلولهای آبی توسط گرانول پوست سخت گردو می باشد. روش بررسی: این تحقیق یک نوع مطالعه بنیادی و تجربی بود که فرآیند جذب بصورت منقطع، در شرایط آزمایشگاهی و با تأکید بر اثر پارامترهای مختلفی چون pH، زمان تماس،غلظت اولیه نیکل و دوز جاذب بر راندمان جذب مورد بررسی قرار گرفت. در هر مرحله از آزمایش‌ها، 100میلی لیتر محلول نیکل با غلظت اولیه مشخص و pH  معین در دستگاه  شیکر بهم زده شد. تأثیر پارامترهای مختلف، (8-2) pH، زمان واکنش(180-10دقیقه)، غلظت اولیه نیکل(100-10میلی گرم در لیتر) و مقدار جاذب(1-1/0گرم) بررسی و  سپس نتایج حاصل با ایزوترم های لانگمویر و فروندلیچ و همچنین سینتیک های شبه درجه اول و شبه درجه دوم برازش شد. یافته‌ها: نتایج مطالعه حاضر نشان داد که حداکثر  کارایی جذب نیکل در محدوده pH برابر با 5 تا 8، میزان دوز جاذب 4/0گرم در 100 میلی لیتر و زمان تماس 90 دقیقه حاصل می‌‌شود. بعلاوه مشاهده گردید که داده‌های جذب از ایزوترم لانگمویر با ضریب همبستگی (99/0R2=) و سینتیک شبه درجه اول (9922/0R2=) و شبه درجه دوم (9937/0R2=) تبعیت می‌کند. نتیجه‌گیری: بر مبنای نتایج حاصل از این مطالعه می‌توان اظهار نمود که گرانول پوست سخت گردو بعنوان یک جاذب طبیعی قادر به حذف مطلوب نیکل از محلولهای آبی می‌باشد و بدلیل توانایی جذب بالا جهت سایر آلاینده‌های زیست محیطی نیز پیشنهاد می‌گردد. این مقاله برگرفته از پایان نامه دانشجویی کارشناسی ارشد دانشگاه علوم پزشکی و خدمات بهداشتی درمانی شهید صدوقی یزد می‌باشد.

Title: بررسی شیوع شپش سر در دانش آموزان مقطع ابتدایی و تعیین کننده های رفتارهای پیشگیری کننده ازآن براساس الگوی اعتقاد بهداشتی در مادران آنهادر هشتگرد، سال تحصیلی92-91

http://tbj.ssu.ac.ir/article-1-1986-fa.html

چکیده مقدمه:آلودگی به شپش سر یک معضل بهداشتی با سابقه طولانی در سطح جهان است که سبب بوجود آمدن عوارض جسمی، روانی و اجتماعی می گردد و در سنین 11-6 سال شایعتر است. نظر به کمبود مطالعات در این زمینه، مطالعه حاضر با هدف بررسی شیوع شپش سر در دانش آموزان مقطع ابتدایی و تعیین کننده های رفتارهای پیشگیری کننده ازآن براساس الگوی اعتقاد بهداشتی در مادران آنهادر هشتگرد، در سال تحصیلی 92-1391 انجام شد. روش بررسی: این مطالعه تحلیلی بصورت مقطعی بر روی372 نفر از دانش آموز مقطع ابتدایی و مادران آنها در شهرستان هشتگرد انجام شد.داده ها به وسیله معاینه سر دانش آموزان و تکمیل پرسشنامه محقق ساخته توسط مادرانشان جمع آوری گردید. تجزیه و تحلیل داده ها توسط نرم افزارSPSS و با استفاده از شاخص های مرکزی و پراکندگی، تی مستقل، ضریب همبستگی پیرسون، رگرسیون خطی وآنالیز واریانس صورت گرفت. یافته ها:میزان شیوع شپش در افراد مورد بررسی 6/4 درصد بود. شیوع آلودگی به شپش سر به طور معنی داری در دانش آموزان دختر(6/8 درصد) بیشتر از دانش آموزان پسر (5/0 درصد) بود(0001/0>P). بین رفتارهای پیشگیری کننده از شپش سر در مادران  و سازه های شدت درک شده، منافع درک شده و خودکارآمدی همبستگی مثبت و معنی دار مشاهده شد(05/0>P).  از بین رفتارهای پیشگیری کننده از شپش سر، همکاری مادران با مراقبین بهداشتی و پرسنل بهداشتی جهت پیشگیری از شپش سر،  کمترین رفتار گزارش شده  بود. سازه های الگوی اعتقاد بهداشتی 7/10 درصد از واریانس رفتارهای پیشگیری کننده  را پیش بینی کردند که تنها قدرت پیش بینی خودکارآمدی به لحاظ آماری معنی دار بود(048/0=&beta;). نتیجه گیری: با توجه به شیوع قابل توجه شپش سر به ویژه در دانش آموزان دختر ، اجرای برنامه های آموزشی مبتنی بر الگوی اعتقاد بهداشتی به منظور بهبود رفتارهای پیشگیری کننده از شپش سر جهت والدین، دانش آموزان و کارکنان مدارس با تأکید بر ارتقاء خودکارآمدی مادران در این زمینه و نیز لزوم همکاری بیشتر والدین و مدرسه در این زمینه پیشنهاد می گردد. این مقاله حاصل از پایان نامه کارشناسی ارشد رشته آموزش بهداشت دانشکده بهداشت دانشگاه علوم پزشکی شهید صدوقی یزد می باشد.

Title: بررسی میزان اثر پوشش خوراکی عصاره دارچین بر مهار رشد آسپرژیلوس فلاووس مولد آفلاتوکسین برروی مغز بادام

http://tbj.ssu.ac.ir/article-1-1987-fa.html

مقدمه: آفلاتوکسین‌ها از شایعترین و خطرناکترین مایکوتوکسین‌ها هستند که توسط قارچهای سمی که بر روی طیف وسیعی از مواد غذایی انسانی و حیوانی رشد میکند تولید میشوند. این گروه از سموم قارچی عوارض و خطراتی از جمله القاء سرطان کبد دارند. با توجه به معایب بعضی از روش های فیزیکی و شیمیایی برای کنترل آلودگی قارچها، اخیرآ توجه زیادی بر روی استفاده از عصاره گیاهان دارویی برای کنترل آلودگی های قارجی مولد آفلاتوکسین بر روی مواد غذایی شده است، لذا هدف از انجام این مطالعه بررسی تاثیر پوشش خوراکی حاوی عصاره دارچین بر روی مهار رشد آسپرژیلوس فلاووس مولد آفلاتوکسین بر روی مغز بادام می باشد. روش بررسی: در این مطالعه مداخله ای تجربی نمونه های مغز بادام را بطور تصادفی  برای انجام مطالعه انتخاب شدند. نمونه های مغز بادام را ابتدا در محلول پوشش خوراکی آب پنیر حاوی3 درصد عصاره هیدروالکلی دارچین بصورت غوطه وری تیمار و سپس با سوسپانسیون 103×1کونیدیهای قارچ آسپرژیلوس فلاووس مولد آفلاتوکسین آلوده شدند. میزان مهار آلودگی مغزهای بادام را با کشت محلول شستشوبادامهای آلوده شده و شمارش تعداد کونیدی های زنده قارچ ارزیابی شدند. در نهایت مقادیر MFC و MIC50 هر یک از عصاره مورد بررسی با تهیه رقتهای سریالی از عصاره ها و آلوده کردن با سوسپانسیون کونید قارچ مطابق با دستورالعمل روش استاندارد CLSI تعیین شدند. یافته ها: در مجموع با مشاهده ماکروسکوپی مغزهای تیمار شده نشان داد که پوشش خوراکی حاوی عصاره 3% هیدروالکلی دارچین توان مهار کامل رشد قارچ مورد بررسی بر روی مغز بادام بود. با انجام تست آماری T زوج تفاوت آماری کاملآ معنی داری بین میانگین تعداد کلنی های جدا شده از کشت محلول شست و شوی مغزهای گروه تست و کنترل بدست آمد (0001/0)p=.  میزان MFC و   MIC50 برای دارچین به ترتیب معادل غلظت های 187 و 94 میلی گرم درصد بدست آمد. نتیجه گیری: نتایج مطالعه حاضر نشان داد که پوشش خوراکی حاوی عصاره دارچین درکنترل رشد قارچ آسپرژیلوس فلا ووس بر روی مغز بادام موثر میباشد. هر چند استفاده عملی از این عصاره ها نیاز مند بررسی های بیشتر اقتصادی، میکروبی و سم شناسی می باشد.

Title: بررسی تاثیر آموزش ذهن‌آگاهی بر رضایتمندی زناشویی واختلال وسواسی- جبری زنان یزد

http://tbj.ssu.ac.ir/article-1-1988-fa.html

مقدمه‌: هدف اصلی درمان شناختی مبتنی بر حضور ذهن آن است که بیمار، از طریق پایش خودایند از اثرات فعالیت مجدد ذهنیت انجامی آگاهی می‌یابد واز طریق ایجاد و به کار گیری حالت حضور ذهن در ذهنیت بودن بماند واز طریق انجام تمرین‌های مکرر با جهت‌دهی قصد مندانه توجه به یک شی خنثی مثلا جریان تنفس به مشاهده افکار و احساسات یا حسهای بدنی خود بیندیشد. هدف این پژوهش بررسی تاثیر آموزش ذهن‌آگاهی گروهی بر رضایتمندی زناشویی و اختلال وسواسی- جبری می‌باشد. روش‌بررسی‌: پژوهش حاضریک مطالعه مداخله ای نیمه تجربی (یک گروهی) می‌باشد.آزمودنی‌های پژوهش با فراخوان تحقیق به روش نمونه‌گیری در دسترس انتخاب شدند سپس فرم کوتاه پرسشنامه رضایتمندی زناشویی اینریچ و پرسشنامه اختلال وسواسی- جبری مادزلی به افراد داده شد. علاوه بر دو ابزار بالینی، با مصاحبه تشخیصی بر اساس(DSM-IV) اختلال وسواسی- جبری آنها ارزیابی شد. ازجامعه زنان متاهل شهریزد که اختلال وسواسی- جبری داشتند و 6ماه بود که تحت درمان دارویی بودند، 30 نفر به صورت تصادفی انتخاب و آزمودنی‌ها در دو گروه آزمایش و کنترل جای گرفتند. گروه آزمایش در 8 جلسه تحت درمان شناختی مبتنی بر ذهن‌آگاهی قرار گرفتند. مداخله آموزشی به مدت 2ماه انجام گرفت، اطلاعات حاصل جمع‌آوری وتوسط نرم‌افزار SPSS با استفاده از آزمون آماری تحلیل کو‌واریانس مقایسه شدند. یافته‌ها: طبق نتایج بدست آمده‌، میانگین نمرات رضایتمندی زناشویی پیش‌آزمون در گروه کنترل وآزمایش به ترتیب برابر با 8354/2 و 1546/3 بوده و در پس‌آزمون به ترتیب 8453/2 و3617/3 بود همچنین میانگین شدت علایم وسواسی- جبری در پیش‌آزمون در گروه‌کنترل و آزمایش به ترتیب5244/0 و 4933/0 بوده و در پس‌آزمون به ترتیب 4866/0 و 3822/0بود. نتیجه‌گیری: این درمان به طور معنی‌داری منجر به افزایش رضایتمندی در گروه آزمایش نسبت به گروه کنترل شده است. در خصوص آموزش ذهن‌آگاهی بر روی کاهش شدت علائم وسواسی- جبری موثر بود ولی رابطه معنی‌دار نبود .

Title: بررسی عوامل مرتبط با ضعف انضباط در رانندگی رانندگان شهر یزد در سال 1393

http://tbj.ssu.ac.ir/article-1-1989-fa.html

مقدمه: حوادث راهنمایی و رانندگی از علل عمده مرگ و میر و جراحات و نیز به عنوان یک مشکل بهداشت عمومی در سطح جهانی شناخته می شوند. از میان عوامل متعددی که در بروز حوادث نقش دارند، رفتار مخاطره آمیز راننده به عنوان شایع ترین عامل ایجاد تصادفات می باشد. مطالعه حاضر با هدف بررسی عوامل مرتبط با ضعف انضباط در رانندگی رانندگان شهر یزد در سال 1393 صورت پذیرفته است. روش بررسی: مطالعه حاضر یک مطالعه مقطعی است که بر روی 373 راننده شهر یزد در سال 1393 صورت پذیرفته است. نمونه گیری با استفاده از روش در دسترس بود. داده ها توسط پرسشنامه محقق ساخته جمع آوری شده و به وسیله نرم افزار آماری 16 SPSS  با استفاده از آزمون های آماری همبستگی پیرسون، آنالیز واریانس یکطرفه، تی مستقل و رگرسیون خطی مورد تجزیه و تحلیل قرار گرفتند. یافته ها: یافته های مطالعه نشان داد میانگین نمره رفتار پرخطر در بین رانندگان از حداکثر 100 نمره،  41/81 بود و نگرش ریسک پذیری و خطر درک شده با رفتار پرخطر رانندگی همبستگی معنی داری داشتند (001/0> p). از بین متغیرهای وارد شده به مدل رگرسیونی نگرش ریسک پذیری و خطر درک شده توانستند 35 درصد تغییرات رفتار پرخطر رانندگان را پیش بینی کنند. (35/0, R2=59 /0, R=2/27F= ). نتیجه گیری: نتایج مطالعه نشان داد رفتارهای پرخطر رانندگی تحت تأثیر عواملی چون نگرش ریسک پذیری و خطر درک شده راننده قرار دارد. بنابراین در نظر گرفتن این عوامل در مداخلات آموزشی ضروری می نماید.
